# Microbial Dispersal, Including Bison Dung Vectored Dispersal, Increases Soil Microbial Diversity in a Grassland Ecosystem

**DOI:** 10.3389/fmicb.2022.825193

**Published:** 2022-03-31

**Authors:** Jaide H. Hawkins, Lydia H. Zeglin

**Affiliations:** Division of Biology, Kansas State University, Manhattan, KS, United States

**Keywords:** soil microbiology, microbial biogeography, grassland management, grazing (rangelands), fire

## Abstract

Microbial communities display biogeographical patterns that are driven by local environmental conditions and dispersal limitation, but the relative importance of underlying dispersal mechanisms and their consequences on community structure are not well described. High dispersal rates can cause soil microbial communities to become more homogenous across space and therefore it is important to identify factors that promote dispersal. This study experimentally manipulated microbial dispersal within different land management treatments at a native tallgrass prairie site, by changing the relative openness of soil to dispersal and by simulating vector dispersal *via* bison dung addition. We deployed experimental soil bags with mesh open or closed to dispersal, and placed bison dung over a subset of these bags, to areas with three different land managements: active bison grazing and annual fire, annual fire but no bison grazing, and no bison grazing with infrequent fire. We expected microbial dispersal to be highest in grazed and burned environments, and that the addition of dung would consistently increase overall microbial richness and lead to homogenization of communities over time. Results show that dispersal rates, as the accumulation of taxa over the course of the 3-month experiment, increase taxonomic richness similarly in all land management treatments. Additionally, bison dung seems to be serving as a dispersal and homogenization vector, based on the consistently higher taxon richness and increased community similarity across contrasting grazing and fire treatments when dung is added. This finding also points to microbial dispersal as an important function that herbivores perform in grassland ecosystems, and in turn, as a function that was lost at a continental scale following bison extermination across the Great Plains of North America in the nineteenth century. This study is the first to detect that dispersal and vector dispersal by grazing mammals promote grassland soil microbial diversity and affect microbial community composition.

## Introduction

Microorganisms are the most diverse group of life on the planet (Locey and Lennon, 2016) and are integral to ecosystem functions such as nutrient cycling, biomass production, and carbon storage (Schimel and Schaeffer, 2012; Colman and Schimel, 2013; Glassman et al., 2018; Kuypers et al., 2018). Yet, a mechanistic understanding of the biogeography of microbial taxa lags behind the extensive research for other organisms, such as plants and animals (Hanson et al., 2012). Accumulated evidence that microbial taxa can be dispersal limited and subject to the legacy of community assembly under historical environmental conditions, as opposed to being globally dispersed and filtered for survival by current local environmental conditions (Martiny et al., 2006; van der Gast, 2015), has created new questions about microbial metacommunity dynamics and the resulting biogeographical patterns that emerge.

Like macro-organismal communities, microbial communities assemble through a combination of dispersal from a regional taxon pool and successful growth in local conditions (Leibold et al., 2004; Hanson et al., 2012; Lindström and Langenheder, 2012; Nemergut et al., 2013). In some cases, the patterns that emerge from these mechanisms are similar to macro-organisms, while in other contexts they are different. For example, microbial taxa often display the same broad spatial scaling patterns that are found among plants and animals (Green and Bohannan, 2006; Locey and Lennon, 2016), but the strength of these patterns can be weaker for microbial life due to biological and methodological differences, such as dormancy and sampling extent (Locey, 2010; Meyer et al., 2018). One biogeographical pattern that exists across macrobial and microbial communities is that of distance-dissimilarity, or the decrease in community similarity with geographical distance (Soininen et al., 2007). The strength of the distance-dissimilarity relationship (i.e., the slope of regression line of community dissimilarity against geographic distance) depends on the balance between two main mechanisms of community assembly—environmental filtering and dispersal limitation (Nekola and White, 1999; Soininen et al., 2007; Hanson et al., 2012). Although environmental filtering has been more thoroughly investigated in microbial communities (Hanson et al., 2012), dispersal limitation can alter metacommunity dynamics by increasing similarity at close locations, while higher dispersal rates can increase similarity at farther locations through mass effects, which weakens the relationship between distance and dissimilarity. It is therefore critical to understand how different environmental attributes affect the interplay of underlying metacommunity assembly mechanisms.

Grasslands are diverse and globally important biomes that provide critical ecosystem services (Bengtsson et al., 2019) and are subject to environmental change due to shifting management practices, such as varying fire and grazing intensities (Bond et al., 2004; Briggs et al., 2005; Borer et al., 2014). How grassland fire and grazing management affects the relative importance of environmental filtering and dispersal limitation in structuring soil microbial communities is still unresolved. In northern China, Cao et al. (2016) determined that environmental filtering, mainly through soil pH and climatic factors, was the main process shaping microbial community distribution, but Richter-Heitmann et al. (2020) found the opposite, that purely deterministic assembly processes could not explain soil microbial diversity in temperate grasslands in Germany and that dispersal was important to both dominant and rare taxon dynamics. Overall, even if the mechanisms are unknown, it is clear that grassland soil microbial communities respond to large ungulate grazing *via* shifts in activity (Esch et al., 2013; Cline et al., 2017; Eldridge et al., 2017) and composition (Patra et al., 2005; Cline et al., 2017). Fire also alters the soil environment *via* direct heat, removal of organic matter, and subsequent changes to soil nutrient availability (Docherty et al., 2012). Although responses vary by grassland site, frequent fire can lead to increased soil microbial activity and shifts in composition (Pérez-Valera et al., 2017; Carson and Zeglin, 2018; Yang et al., 2020). These and many more studies demonstrate the importance of environmental filtering on grassland soil microbial communities, but dispersal could also have important consequences for microbial community assembly.

Dispersal is the least understood microbial community assembly mechanism in most ecosystems (Albright and Martiny, 2018), but could be affected by fire and grazing. In all grasslands, grazing is the critical ecological and evolutionary interaction between large herbivores and dominant plants (Stebbins, 1981). Before European settlement and “systematic slaughter” (in the words of Hornaday, 1889) of bison across the Great Plains, reducing the population to an estimated hundreds of animals by the end of the nineteenth century, bison numbered an estimated 25–30 million, and their range spanned more than a third of the continent (Lueck, 2002). Bison are particularly integral for North American tallgrass prairies as they hold a keystone role historically and contemporarily, increasing plant diversity, soil fertility, and forage quality in their zones of influence (Knapp et al., 1999). At sites across the Great Plains, bison grazing tends to decrease the strength of the soil microbial distance-dissimilarity relationship (Allenbrand, 2020), and bison reintroduction to Tallgrass prairie can cause convergence of soil microbial communities with varied management backgrounds, with their dung implicated as an important mechanism for this homogenization (Chantos, 2017). Also, North American bison have a distinct gut microbiome (Bergmann et al., 2015), as do most megaherbivores in more ancient grasslands (Kartzinel et al., 2019). Therefore, as large herbivores like bison move around the landscape, they may serve as vectors to disperse microbial cells *via* dung deposition. Concurrently, dispersal of microbial cells *via* aerial deposition and through water films in soil pores is also likely (Finlay and Clarke, 1999; Bottos et al., 2014; Yang and van Elsas, 2018; Elliott et al., 2019), and fire and grazing could influence how readily airborne cells reach the soil, since both create bare soil patches open to aerial inputs (Bakker et al., 2003; Henry et al., 2006). Further, fire can promote aerial dispersal of microbes, by aerosolizing viable soil microbial cells and spores (Kobziar et al., 2018; Moore et al., 2020). In sum, both bison grazing and fire could increase dispersal of soil microorganisms: Bison as a vector of dispersal, and fire as a direct vector, or non-vector mechanism that increases soil exposure to aerial dispersal.

To measure the degree to which non-vector and vector dispersal mechanisms are operating, it is necessary to experimentally alter the dominant factors predicted to be responsible for the pattern (Green and Bohannan, 2006). Two main research approaches, excluding modeling, can be taken for experimental evaluation of assembly mechanisms: environmental manipulation or altering dispersal rates (Hanson et al., 2012). Studies that manipulated microbial dispersal have been successful in altering rates and composition of dispersed taxa, and showed that altered dispersal has a significant effect on community dynamics (Bell, 2010; Berga et al., 2015; Albright and Martiny, 2018). Therefore, we designed an experiment to learn how environmental manipulation (*via* differences in fire and grazing management), and alteration of dispersal rates (*via* manipulation of soil openness to aerial dispersal, and active addition of bison dung as a dispersal vector) influence dispersal limitation and subsequent assembly dynamics of soil microbial communities in a grassland ecosystem.

**FIGURE 1:**
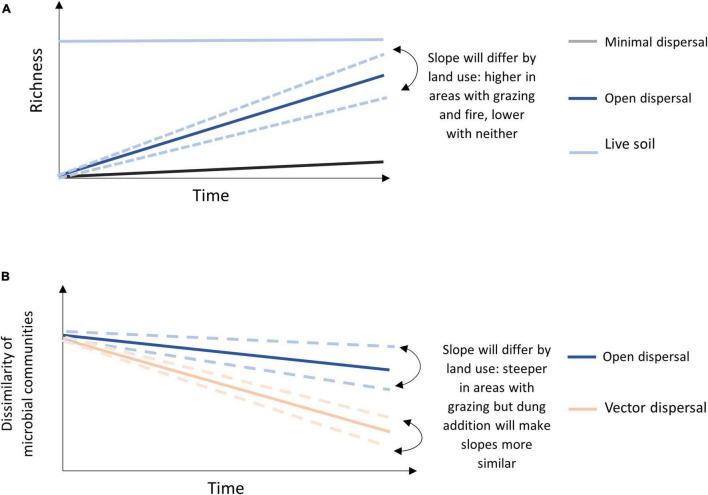
Conceptual model of **(A)** predicted OTU accumulation over time for each treatment with indication of land management effect for the open sterile slope, and **(B)** predicted microbial community dissimilarity of open dispersal treatment and vector dispersal treatment (dung amended sterile soil) across land management treatments over time.

We hypothesized that dispersal of microbial taxa would be higher in burned and grazed areas than in unburned and ungrazed areas, but that burned area (open canopy) communities would display more heterogeneous assembly from aerial dispersal, while communities in grazed areas would converge in community composition due to bison-vectored dispersal (through bison dung). To test the hypotheses, we manipulated the potential rate of passive dispersal using soil bags with open or closed mesh (Albright and Martiny, 2018), and manipulated active dispersal using addition of fresh bison dung to sterilized and non-sterilized (“live”) soil, and deployed these experiments in replicates across grazed, burned, and neither grazed nor burned watersheds at the Konza Prairie Biological Station (KPBS, Manhattan, KS, United States). Specific predictions included: (1) dispersal rates, or accumulation of new microbial taxa over time in sterilized soil open to dispersal, will occur in all conditions but will be highest in burned areas and lowest in unburned and ungrazed areas, and (2) vector dispersal, *via* the addition of bison dung, will increase the number of new microbial taxa in all environments, and also lead to microbial community convergence regardless of fire or grazing conditions (Figure 1).

## Materials and Methods

### Study Location

This experiment was performed at Konza Prairie Biological Station (KPBS), located in northeastern Kansas, United States (39°05′N, 96°35′W), part of the Flint Hills region of KS and OK, one of the few, and largest, remaining native tracts of tallgrass prairie. KPBS was established as a research station in 1971, and became host to a Long-Term Ecological Research (LTER) project in 1980. Watershed scale treatments of differing fire intervals have been in place since the 1970s, and bison were reintroduced to a subset of these watersheds in the late 1980s—early 1990s, thus large areas with contrasting land management treatments have been maintained for decades. For this study, experimental research was restricted to upland soils (Florence series, Udic Argiustolls) in three of the environmental treatments: ungrazed and infrequently burned (20 year fire interval), bison-grazed and infrequently burned, and ungrazed and frequently burned (annual fire interval). No infrequently burned watersheds experienced fire in the study year, so are referred to as “unburned” treatments hereafter.

### Experimental Design

**FIGURE 2:**
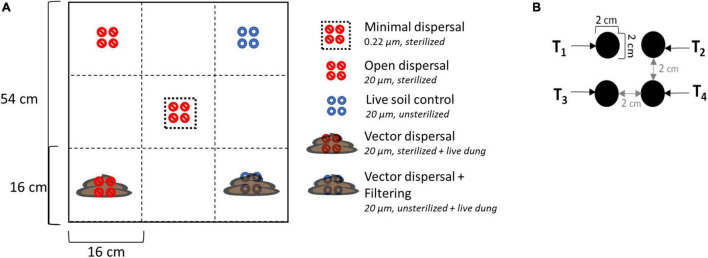
**(A)** Layout of experimental unit with treatments randomly assigned in a checkerboard pattern. There were four replicates in each of the three land management treatments. The legend also displays the mesh bag pore size and sterilization status of the soil inside the bag. Live = unsterilized **(B)** enlarged diagram of individual treatment layout; each treatment has four individual soil bag samples corresponding to sampling time points.

Dispersal manipulations were installed across the experimental landscape, with four field replicates in each of the three different land use treatments. Each experimental unit contained five different dispersal treatments randomly assigned in a checkerboard pattern: sterilized soil closed to dispersal (minimal dispersal), sterilized soil open to dispersal (open dispersal), live soil open to dispersal (live soil control), sterilized soil open to dispersal from bison dung (vector dispersal), and live soil open to dispersal from bison dung (vector dispersal + filtering) (Figure 2). The open vs. closed dispersal contrast was achieved using nylon mesh (Tisch Scientific, North Bend, OH, United States) bags with pore sizes of 20 and 0.22 μm, respectively. These mesh sizes have been shown to successfully manipulate bacterial and archaeal migration rate (Albright and Martiny, 2018). The soil inside the bag was either live (unsterilized) or sterilized (*via* autoclaving at 121°C degrees for 20 min), and bags were always deployed to the same land use from which the soil was collected, to remove any confounding soil physicochemical environmental effects. In the vector dispersal treatments, recently collected fresh bison dung was deposited on top of the live and sterile soil bags after the bags were placed into the ground. Each treatment included four bags that were collected 1 day (T1), 1 week (T2), 1 month (T3), and 3 months (T4) post deployment, plus subsampling of the overlying bison dung at each time point. With 6 sample types (5 treatments + subsampling of dung), 3 land use treatments, 4 time points, and 4 replicates, 288 samples were collected in total.

### Treatment Preparation and Installment

Fresh bison fecal samples were collected into gallon Ziploc bags using aseptic technique on 4 June 2019. Areas of the dung touching soil or vegetation was avoided, and dung was only collected from bison 2 years and older to ensure they had weaned and were eating a representative diet. The samples were kept on ice until transported back to the lab where they were stored at −20°C until further analysis. A subsample of approximately 50 mL was retained for reference data collection, and the remaining dung was divided in half to process for treatments.

Experimental unit locations were established and soil for the dispersal bags was collected from each sampling point within the unit using a 2 cm diameter soil auger to a depth of 2 cm. Soils were homogenized into one composite sample for each land use treatment, and plant material was removed, by sieving through 4 mm mesh using aseptic technique. A subsample for live soil and sterilized soil from each of the land use treatments was collected and stored at −20°C for characterization of initial soil microbial communities as a reference.

Open and closed dispersal soil bags were made with two different materials: nylon mesh with a pore size of 20 μm and a nylon membrane mesh with a pore size of 0.22 μm, respectively. Each bag had a dimension of 2 cm × 2 cm, but open and closed bags were constructed with two different methods. Using aseptic technique, the open bags were sewn using weather resistant nylon thread stitched along three edges with a folded edge to decrease the amount of stitching. Using aseptic technique, the closed bags were glued using Gorilla Glue Clear Grip along three edges with a folded edge. A small opening was left in each bag for filling, which was then closed with the corresponding method and bags were further processed according to dispersal treatment. All bags used for live soil treatments were sterilized by autoclaving prior to filling and closed using aseptic technique and placed in UV-sterilized 1-L Nalgene bottles according to land use history. All bags used for sterile soil treatments were sterilized after filling by placing in UV-sterilized 1-L Nalgene bottles according to land use history and autoclaved with the lids loosely on. This allowed for aseptic transport to the field site for installation.

Soil bags were deployed back into sampling locations according to land use treatment. Live bison dung was deposited in equal amounts on top of soil bags according to treatment assignment. All soil bags and dung were placed underneath any surface litter that was present. At each sampling time point, the appropriate soil bag was extracted, transported to the lab, soil was transferred from the nylon bags to pre-labeled gamma-sterilized centrifuge tubes for storage at −20°C until further processing.

### DNA Extraction and Polymerase Chain Reaction

Total genomic DNA (gDNA) was extracted from approximately 0.5 g of homogenized soil or dung per sample using the Qiagen DNeasy PowerSoil kit (Qiagen Sciences, Germantown, MD, United States) following manufacturer’s instructions but with the following modifications: PowerBead Tubes were disrupted by bead beating for 20 s using a MP Biomedicals (Santa Ana, CA, United States) sample disruptor set at 4 m/s velocity, supernatant was transferred using the recommended minimum volume, and for final DNA elution step 50 μL of solution C6 was added and incubated for 5 min at room temperature before spinning down and repeated using the flow-through. In addition, since dung collected at later time points was markedly more desiccated and thus absorbed water, 500 μL of extraction buffer was added to the PowerBead column and then filled to capacity with dung even if dung mass was below 0.5 g. Genomic DNA (gDNA) was stored at −20°C until further analysis. Yield of gDNA was measured using a ThermoFisher Quant-iT PicoGreen dsDNA Assay Kit and quantified gram^−1^ dry soil (Thermo Fisher Scientific Inc., Waltham, MA, United States).

From the gDNA extracts, the bacterial and archaeal 16S rRNA gene was targeted for Illumina sequencing using universal primers (515F/926R) following established protocols (Caporaso et al., 2012; Parada et al., 2016) with one modification: Polymerase Chain Reaction (PCR) was run for 25 cycles instead of 35. Three technical replicates were run for each barcoded sample and reaction success was confirmed with 1% agarose gel electrophoresis. Upon successful PCR, technical replicates were pooled, cleaned using Exo-SapIT (Applied BioSystems, Foster City, CA, United States), and amplicon pools quantified using the Quant-iT PicoGreen assay kit (Life Technologies, Grand Island, NY, United States). Amplicon amounts were then normalized to 75 ng per barcoded sample, combined into one library and cleaned using a QIAquick Gel Extraction Kit (Qiagen, Germantown, MD, United States). The library was sequenced on a 2 × 250 paired-end read Illumina MiSeq run with 15% PhiX at the Kansas State University Integrated Genomics Facility.

### Bioinformatics

Raw Illumina sequence data was processed using the QIIME2 software package (Bolyen et al., 2019). Sequences were demultiplexed and joined. Proceeding with only the forward reads, sequences were quality controlled and chimeras removed using Dada2 with default parameters, where reads truncated at the first instance of a quality score less than or equal to two (Callahan et al., 2016). The remaining sequences were clustered to 97% sequence similarity and assigned to operational taxonomic units (OTUs) using the open-reference workflow. OTUs were aligned to the GreenGenes (DeSantis et al., 2006) v 13.18 16S rRNA gene reference database and taxonomy assigned using a Naïve-Bayes classifier (Pedregosa et al., 2011) trained at 97% similarity. Singletons and doubletons (as per the rare feature cutoff threshold recommended in Bokulich et al., 2013), chloroplast sequences, and mitochondrial sequences were removed using filter functions before further analysis.

The remaining pre-processing, statistical analysis and visualizations were performed in R version 3.6.2 (R Core Team, 2019). The sequence library was further processed using phyloseq version 3.10 (McMurdie and Holmes, 2013) by creating a phyloseq object and removing samples that did not have at least 3,000 reads, resulting in a dataset with 272 samples and 6,500,692 total sequences with 15,326 unique OTUs. From this, two separate datasets were created: a rarefied dataset with all samples trimmed to 3,000 sequences by random sampling resulting in 272 samples and 816,000 total sequences with 11,664 unique OTUs, and a normalized data set by proportional transformation of each sample using total sequence counts resulting in 272 samples and 2,720,000 total sequences with 15,326 unique taxa. The low sequence count for the rarefied dataset was selected as the best approach to retain as many low-diversity samples as possible from the early experimental time points (Supplementary Figure 1).

### Dispersal Analysis

All alpha diversity metrics were calculated using “phyloseq” and the statistical testing was done with base R and “vegan” (Oksanen et al., 2019). The alpha diversity metric of observed OTUs was calculated using the rarefied dataset with the estimate_richness function from “phyloseq” (McMurdie and Holmes, 2013). To test the effect of dispersal treatment, fire and grazing treatment, and the interaction between the two, on DNA yield and microbial richness for all time points, we used two-way analysis of variance (ANOVA) models and lsmeans function for *post hoc* pairwise comparison of groups. To test the effect of dispersal and land use treatment on DNA yield and microbial richness as a function of time, we used general linear models to perform analysis of covariance (ANCOVA) with the lm function (R Core Team, 2019) and Anova function (Fox and Weisberg, 2019). Response variables were assessed for normality, and only DNA yield needed to be log transformed to normalize data distribution prior to parametric statistical analysis. The models tested time, treatment, land use history and the pairwise interactions between all three (time by dispersal treatment, time by land use, dispersal treatment by land use, and the three-way interaction). Pairwise ANCOVA models were performed on different treatment subsets to evaluate specific predictions regarding the effect of dispersal treatment and land use treatment on dispersal and richness (Figure 1A). For significant effects, least square means for all pairwise comparisons were used as the *post-hoc* test for identification of significantly different levels using the lsmeans function (“lsmeans,” Length, 2016) and cld function (“multcompView,” Graves et al., 2019).

### Community Composition

A Bray-Curtis dissimilarity matrix was calculated from the normalized dataset to evaluate beta diversity, and community differences were visualized with non-metric multidimensional scaling (NMDS). A 3-way permutational multivariate analysis of variance (PERMANOVA) was used to evaluate the effect on community composition of dispersal treatment, fire and grazing treatment, time, and their interaction using the adonis function in vegan with 999 permutations (Oksanen et al., 2019). To qualitatively evaluate community dispersion of dispersal treatments, the betadisper function in vegan was used to calculate the sample distance to group centroid of the Bray-Curtis dissimilarity matrix, with groups defined as dispersal treatments at each time point. The average distances were then plotted as function of time in days using ggplot, and used as the response variable to run ANCOVA to test the effects of dispersal treatment and time using the lm and anova functions. Least square means for all pairwise comparisons were used as the *post-hoc* test for identification of significantly different levels using the lsmeans and cld functions.

## Results

### DNA Yield

**FIGURE 3:**
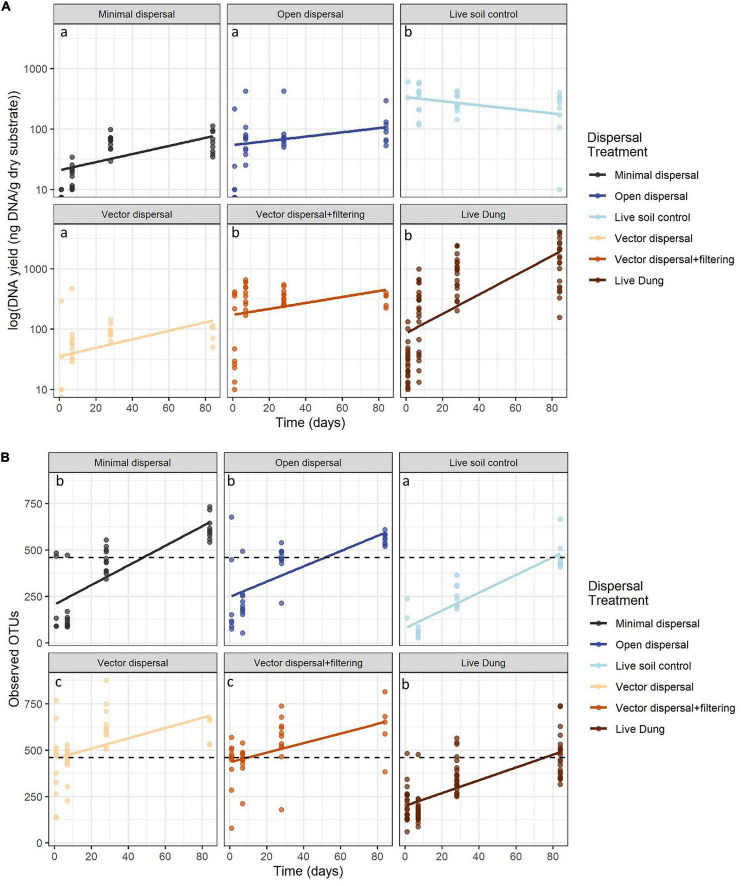
**(A)** DNA yield (ng g-1 dry substrate) across time and **(B)** microbial richness (observed OTUs) across time with reference soil richness indicated by black dashed line. Ordinary least squares regression lines displayed and colored by treatment, and *post hoc* groupings for the intercept are indicated by lower-case letters.

In all sterile soil treatments, DNA yield was undetectable at time zero, and increased significantly over the incubation period (Figure 3A and Table 1). Throughout the experimental time-series, DNA yield remained lower in the minimal dispersal (0.2 μM mesh) treatment relative to the open dispersal (20 μM mesh) treatment, higher in vector-dispersal soil treatments than the open dispersal soil treatment, and higher in the live dung than the vector-dispersal soil treatments (Table 2). DNA yield did not change significantly through the sampling times for the live (unsterilized) soil treatments, but did increase significantly over time in live bison dung (Tables 1, 2 and Figure 3A). Land-use treatment effects were not significant (Table 2 and Supplementary Figure 2A).

**TABLE 1:** Slopes and intercepts for full linear models for each experimental dispersal treatment pooled across land management types; model = log (DNA yield + 1) or OTU richness ∼ Time in days *Treatment.

	DNA yield			OTU richness		
Dispersal treatment	Slope,	Intercept,	*Post hoc* groups	Slope,	Intercept,	*Post hoc* groups
	*p*-value	*p*-value	for intercept	*p*-value	*p*-value	for intercept
Minimal dispersal	**0.02**	**11.9**	a	**5.3**	**205.5**	b
	**0.0002**	**<0.0001**		**<0.0001**	**<0.0001**	
Open dispersal	**0.02**	**28.7**	a	**4.1**	**247.3**	b
	**0.018**	**<0.0001**		**<0.0001**	**<0.0001**	
Live soil control	−0.01	**335.2**	b	**4.8**	**78.3**	a
	0.062	**<0.0001**		**<0.0001**	**0.0007**	
Vector dispersal	**0.02**	**26.7**	a	**2.8**	**453.3**	c
	**0.01**	**<0.0001**		**0.007**	**<0.0001**	
Vector dispersal + filtering	0.01	**173.6**	b	**2.6**	**434.9**	c
	0.092	**<0.0001**		**0.003**	**<0.0001**	
Live dung	**4.48**	**86.8**	b	**3.4**	**199.9**	b
	**<0.0001**	**<0.0001**		**<0.0001**	**<0.0001**	

*Bolded values are significant. Post hoc groups for y-intercepts were defined using P < 0.05 significance threshold for least-squared means among group comparisons.*

**TABLE 2:** ANCOVA results for DNA yield (g g^–1^ dry substrate) and microbial richness (observed OTUs) for models comparing the treatment levels A: minimal dispersal, open dispersal; B: open dispersal, live soil control, vector dispersal, and vector dispersal + filtering; C: vector dispersal, vector dispersal + filtering, and live dung.

	A: Minimal vs. open dispersal		B: Open vs. vector dispersal		C: Live dung vs. vector dispersal	
Factors	DNA yield	Richness	DNA yield	Richness	DNA yield	Richness
	F, P	F, P	F, P	F, P	F, P	F, P
Time	**6.04, 0.017**	**28.4, < 0.0001**	0.996, 0.32	**19.44, < 0.0001**	3.225, 0.075	**3.958, 0.049**
Dispersal treatment (Trt)	**4.4, 0.04**	0.0723, 0.800	**6.23, 0.0006**	**11.704, < 0.0001**	**4.92, 0.009**	**15.136, < 0.0001**
Land Mgt	0.925, 0.402	2.29, 0.11	0.322, 0.756	2.159, 0.12	1.238, 0.293	1.110, 0.332
Time*Trt	0.875, 0.353	0.143, 0.707	1.605, 0.192	1.3, 0.278	1.716, 0.183	0.212, 0.81
Time*Land Mgt	0.002, 0.998	1.04, 0.360	0.642, 0.528	0.697, 0.5	0.188, 0.829	0.44, 0.645
Trt*Land Mgt	0.657, 0.522	2.59, 0.083	0.737, 0.620	1.254, 0.284	1.183, 0.321	1.597, 0.178
Time*Trt*Land Mgt	0.286, 0.752	1.076, 0.348	0.314, 0.929	0.337, 0.916	0.53, 0.713	0.018, 0.999

*Statistical results with P < 0.05 are shown in bold.*

### Richness

Microbial richness increased over time in all experimental dispersal treatments but was not affected by grazing or fire land-use treatment (Figure 3B, Supplementary Figure 2B, and Tables 1, 2). While all slopes of OTU accumulation over time were similar, suggesting a dispersal rate of approximately 2–5 OTUs per day in all experimental treatments, there were significant differences in the intercepts of each model, indicating dispersal treatment effects on total richness that manifested early in the experimental time series (Table 1). The live soil control bags had lowest richness initially, and only reached the level of richness measured in intact field soil reference samples at the final, 3-month, sampling time (Figure 3B). In contrast, the sterilized soil treatments exposed to open dispersal accumulated higher richness in the first week of the experiment, and reached initial field soil reference levels after approximately 3 weeks (Figure 3B and Table 1). The vectored dispersal of microbes in live bison dung increased richness by hundreds of OTUs immediately, an effect that persisted for the duration of the experiment, maintained the highest richness overall, and weakened the slope of OTU accumulation over time (Figure 3B and Tables 1, 2). Also, pure live dung had fewer observed OTUs than the soils with dung added (Figure 3B and Tables 1, 2).

### Community Composition

**FIGURE 4:**
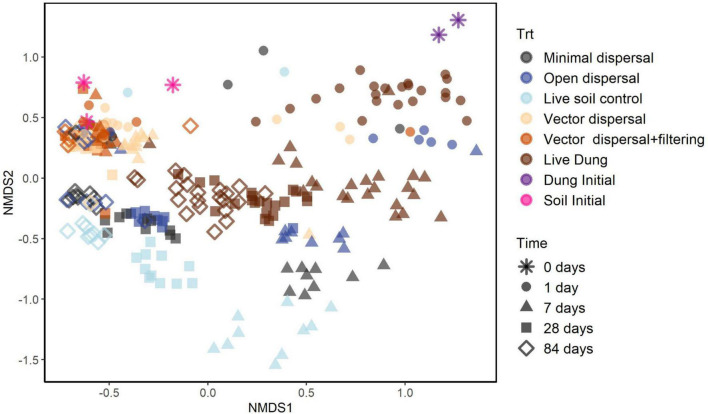
NMDS ordination models of 16S rRNA gene community composition for all samples with colors representing experimental dispersal treatment (including reference soil and dung samples) and symbols representing sampling time.

**FIGURE 5:**
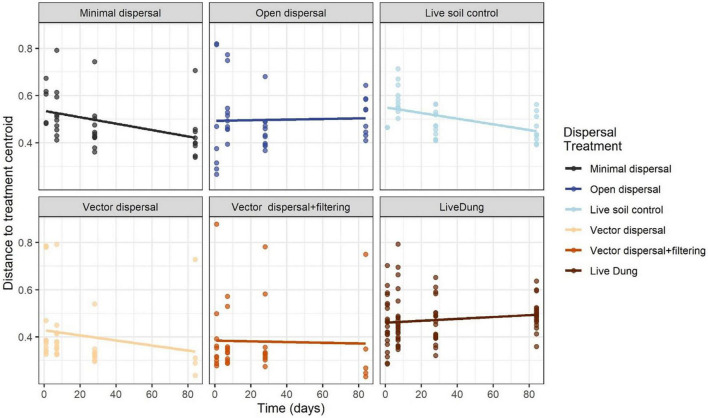
Average distance to group centroids in multivariate space using the Bray-Curtis distance matrix across time for each dispersal experimental treatment. Values closer to 0 indicate less compositional variance across samples within that group, and therefore less dispersion.

Soil microbial community composition was affected significantly by all treatments and their interactions (PERMANOVA, Table 3), with the highest amounts of variation explained by dispersal treatment (22.2%), time (10.1%), and the time by dispersal interaction (12.3%). In the NMDS ordination of all data, community composition shifted temporally, and soil and dung effects were also clearly separated (Figure 4). The community composition of vector dispersal treatments (soils with dung added) converged with the soil reference communities rapidly (after 1 day for the live soils, and after 1 month for the sterilized soils), while the open dispersal treatment communities became more similar to the reference soil communities over time, but did not converge after 3 months. The dispersion around the group centroid of vector dispersal microbial communities was consistently smaller than that of the open dispersal treatment communities without dung addition, and this difference was still apparent at 3 months (*F* = 5.3, *P* = 0.00011; *post-hoc P* < 0.05; Figure 5). There was no consistent trend in the compositional variance of the open treatment communities, while there was a steady decline in the minimal dispersal treatments over time (*F* = 4.6, *P* = 0.034; Figure 5).

**TABLE 3:** PERMANOVA results across all dispersal treatments (Trt), time points (Time), and land management types (Land Mgt) for soil microbial community composition.

Factor	Sum of squares	*F*	*R* ^2^	*P*
Time	11.0	16.7	0.101	0.001*
Trt	24.2	15.7	0.222	0.001*
Land Mgt	2.3	5.2	0.021	0.001*
Time*Trt	13.4	4.1	0.123	0.001*
Time*Land Mgt	1.8	1.4	0.017	0.007*
Trt*Land Mgt	5.9	2.2	0.054	0.001*
Time*Trt*Land Mgt	7.3	1.1	0.067	0.027*

Statistical results with P < 0.05 are shown with an asterisk (*).

## Discussion

This study shows that dispersal generally, including bison-dung vectored dispersal specifically, has a significant influence on soil microbial richness and composition. The experiment revealed that taxon accumulation over time *via* dispersal from aerial or proximate soil sources occurred under all treatment conditions, though fire and grazing management did not have the impact on dispersal rates that we predicted (Figure 3B, Supplementary Figure 2, and Table 2). Beyond the accumulation of taxa through non-vector dispersal, the dispersal through bison dung to both sterile and live soil resulted in an additive effect of hundreds of taxa, which may have contributed to community convergence over time (Figure 5).

It proved difficult to cut off microbial dispersal completely, as DNA and microbial taxa accumulated even in the “closed” (0.2 μM mesh) bags, despite DNA and richness levels below detection at the beginning of the experiment in that treatment, which indicate the pre-experimental sterilization was successful. While the minimal dispersal treatment did not prevent microbial colonization, it did appear to successfully decrease the load of cells that were able to colonize and grow, since DNA levels remained lower through the experiment than the sterilized but “open” dispersal (20 μM mesh) bags (Table 1), which could in part be due to the smaller mesh size restricting dispersal to taxa with cell sizes smaller than 0.2 μM. Colonizers within or after 24 h of exposure could establish if carbon and nutrient sources left after the sterilization death of pre-existing microbial populations provided better environmental conditions for activity and growth. Additionally, dormant taxa in the form of spores or cysts may have survived sterilization and left the dormant state due to more favorable conditions (Locey, 2010), or traces of relict DNA may remain after sterilization (Carini et al., 2016). The slope of richness over time was significantly positive for all dispersal treatments (Figure 3B) and the trajectory of community change over time was similar (Figure 4), so while the DNA load in minimal dispersal bags tended to be lower than that of the open dispersal treatments (Figure 3A), the diversity of the source pool of colonizers appeared similar across all treatments and experimental units, which could point to the dispersal of the same small taxa or growth of the same dormant taxa across all of the sterilized treatments regardless of experimental bag mesh size.

We found non-vectored dispersal to be important across all land use types and evident within 24 h, showing that dispersal is an important contributor to soil microbial richness and composition, and partly supporting our first prediction. Dispersal routes include aerial movement from wind and rain (Bottos et al., 2014) or active movement through the soil matrix (Yang and van Elsas, 2018), with both likely happening in our system. Microbial cells can be transported *via* wind-blown dust at local and regional scales, with distance traveled dependent on wind direction, speed, and soil type (Sabacká et al., 2012; Acosta-Martínez et al., 2015; Elliott et al., 2019). The Great Plains are persistently windy, so it would not be unexpected for aeolian deposition to move microbes around the landscape. Secondly, microbial cells can move within the soil through water-filled pore spaces, which could result in dispersal to neighboring soil locations when water content is sufficiently high (Carson et al., 2010; Kravchenko et al., 2013; Yang and van Elsas, 2018). Movement of microbes within soil can also be driven by biotic interactions, as bacterial cells have been shown to use fungal hyphae as “highways” to navigate the soil matrix (Furuno et al., 2010; Warmink et al., 2011). More experimentation would be needed to parse contributions from these different mechanisms.

Contrary to our predictions, however, landscape-scale bison grazing and fire management treatments did not mediate microbial dispersal effects (Figure 4 and Table 3). The lack of dispersal differences could be because very local scale effects, such as soil openness to dispersal (Albright et al., 2019) and influx of microbial populations from neighboring (sub-centimeters) soil and dung, might matter more than watershed scale environmental factors for overall dispersal rates. Alternatively, the effects of fire and grazing on dispersal might shift with time and our 3-month experiment may not have been long enough to capture this temporal variation. For example, transiently high aerial dispersal rates may have occurred immediately after spring burning, when soil was most exposed and more aerosolized cells were mobile (Kobziar et al., 2018), combined with higher dispersal impact in spring when soil had lower microbial biomass (Wang et al., 2012) and lower plant canopy cover. Our experiment was installed in early June, about 6 weeks after the annual fire; by this time, the peak influence of aerial dispersal might have passed. Also, partially burying the soil bags means that proximate soil communities may have been the dominant source of dispersal, rather than the aerial modes that underlaid our mechanistic predictions about fire and grazing effects. A follow-up dispersal experiment would need to be extended in time, and more explicitly measure aerial inputs of cells, to better evaluate the mechanisms of fire and grazing management on wind or rain driven dispersal.

Bison dung addition, mimicking vector dispersal by grazing ungulates, consistently and substantially increased soil microbial richness and changed the community composition (Figures 3B, 4 and Table 1). Thousands of bacterial and hundreds of archaeal taxa have been identified in bison fecal samples (Bergmann et al., 2015), so it is no surprise that dung may be an important vector of microbial dispersal. Further, a field bison dung incubation experiment conducted at a different tallgrass prairie site observed increased similarity among soil microbial communities after 3 weeks of exposure to the dung (Chantos, 2017). Following dung deposition, microbial dispersal to surface soil could also result from increased activity of dung-affiliated invertebrates, such as dung beetles (Slade et al., 2016), which may move their own host-associated microbiomes in and around the bison dung. Dung beetle abundance and diversity increases with bison presence and recent fire (Barber et al., 2017), and we anecdotally observed dung beetle activity, though we did not measure it. We also did not measure soil nutrient changes during this experiment; however, it is likely that dung quickly leaches labile nutrients and particulate organic matter, enhancing the fertility of the soil below and around it (Johnson and Matchett, 2001; Sitters and Olde Venterink, 2015), before it desiccates and hardens over the weeks of incubation. However, another study saw no effect of bison dung addition on the C:N status of adjacent soil (Chantos, 2017), and therefore the role of environmental filtering through the fertilization effects of dung remains unclear. In our experiment, the dung addition clearly increased the number of microbial taxa in both sterilized and live soil relative to the corresponding treatments with no dung, but dung addition did not shift the live soil community composition toward that of pure dung. Rather, dung-sourced communities became rapidly more similar to soil (Figure 3A and Table 1). Also, as predicted, soil microbial communities converged more strongly and quickly with bison dung addition than with no dung (Figure 5). Thus, while the evidence that dung drives direct dispersal of microorganisms is clear, and it seems likely that the soil’s distinct physicochemical habitat acts as an environmental filter for dung-sourced microorganisms, we still cannot infer the extent to which carbon and nutrients added through dung (Sitters et al., 2014) promoted the colonization and growth of certain taxa.

Unexpectedly, the live soil control experimental treatments had the lowest richness throughout the experiment, substantially less than the intact soil reference samples or the sterilized dispersal treatments (Figure 3B and Table 1). This suggests that removal of the soil from the field for experimental bag construction changed the microbial community, and that the 20 μM mesh barrier prevented the experimentally manipulated soils from recovering to a reference state (Figure 4). Laboratory processing of the soil to set up experimental soil bags could have killed certain taxa, providing the taxa remaining in the live soil control a competitive advantage over dispersers in the field due to earlier access to remaining nutrients, thus establishing communities with lower richness (Mouquet and Loreau, 2002; Svoboda et al., 2018). Such a priority effect is further supported by the combined observations of the live soil control treatments having the greatest DNA yield at the beginning of the experiment, but the lowest richness, indicating dominance of specific taxa that may have changed the community trajectory of these samples (Debray et al., 2022). The absence of this pattern in the initially live soil with dung addition could be because dung also serves as a nutrient source that alleviates resource scarcity, allowing dispersers a better chance of survival. Furthermore, biotic interactions are likely important for microbial community assembly, such that modification or suppression of interactions limits microbial richness in our experiment. For example, predation, which has been shown to increase microbial richness by reducing the survival of dominant taxa and allowing more rare or subordinate taxa to survive (Saleem et al., 2012; Jiang et al., 2017), would be minimal even in the “open” mesh experimental bags. Additionally, competition and cooperation with plant roots (Berg and Smalla, 2009; Haichar et al., 2014), fungi (Deveau et al., 2018), and invertebrates (Wardle, 2006; Bray et al., 2019) are well known biotic factors structuring microbial communities. Roots, invertebrates, and any organisms larger than 20 μm would have been unable to disperse into treatment bags, thus removing important multi-trophic interactions. The artificial conditions imposed by the dispersal experiment, in combination with the low rarefaction threshold required to fairly compare richness numbers among all samples (Supplementary Figure 1), emphasize that this work cannot be used to quantitatively predict *in situ* soil microbial richness levels.

The dual role of long-distance aerial and short-range within soil dispersal makes identifying a regional vs. local signal challenging, but nonetheless, a constant rate of passive dispersal could maintain higher soil diversity across the landscape in a relatively stochastic manner. In metacommunity theory, mass effects—the constant immigration of individuals because of high dispersal rates—can spatially homogenize communities and maintain the presence of rare taxa in communities (Leibold et al., 2004; Lindström and Langenheder, 2012). In microbial communities this effect might be stronger because of microorganisms’ ability to enter dormancy and effectively serve as a “seed bank” if dispersed into initially unfavorable conditions (Locey et al., 2020; Wisnoski et al., 2020). In the context of grassland soil microbial community assembly, successful microbial passage through the ungulate digestive tract, either through dormancy or through facultatively anaerobic growth, serves as a strong filter antecedent to dispersal in dung. Before European colonization of the continent, bison migrated thousands of kilometers in mind-bogglingly high numbers across the North American Great Plains (Knapp et al., 1999). The global decline and extirpation of herbivore populations has detrimental consequences on many ecosystem attributes (Young et al., 2016). The extermination of bison from North America may have removed an important consumer-driven nutrient recycling function (Sitters and Olde Venterink, 2015) across Great Plains grasslands, and our results also suggest the likely loss of an important microbial dispersal mechanism that could impact soil microbial structure and function at both regional and local scales.

Overall, this experiment provides strong evidence that soil microbial dispersal is happening throughout the growing season in both grazed and burned land management environments in tallgrass prairie. Furthermore, vector dispersal through bison dung increases soil microbial community richness and homogenizes composition. Microbial dispersal has real and important consequences on community composition (Albright and Martiny, 2018) and function (Mallon et al., 2015; Evans et al., 2020), knowledge of which could be used to improve ecosystem management, conversation, and restoration. While the same mechanisms drive community assembly for all organisms, different biogeographical patterns may manifest due to the different scales at which these mechanisms act on microorganisms, making prediction of microbial structure and function less reliable when these different scales of influence are not taken into consideration. In this case study, bison’s massively important historical role in grassland soil microbial community assembly, *via* dung-vectored dispersal, could be categorized as regional-scale deterministic mass effects, a category of influence that is not usually considered in metacommunity conceptual frameworks. A large number of contemporary studies on grassland soil microbial ecology are missing this factor, due to a lack of ungulate grazers within the study system. Our results contribute to both an increased understanding of grassland soil microbial community dynamics, and to a growing body of literature on soil microbial biogeography.

## Data Availability Statement

The data presented in this study can be found at the NCBI SRA database under Study PRJNA808890.

## Author Contributions

LZ conceived the initial study idea and edited the final manuscript. JH and LZ designed the study, performed bioinformatics, and contributed to data interpretation. JH carried out field and laboratory work, performed statistical analysis, and drafted the manuscript. Both authors contributed to the article and approved the submitted version.

## Conflict of Interest

The authors declare that the research was conducted in the absence of any commercial or financial relationships that could be construed as a potential conflict of interest.

## Publisher’s Note

All claims expressed in this article are solely those of the authors and do not necessarily represent those of their affiliated organizations, or those of the publisher, the editors and the reviewers. Any product that may be evaluated in this article, or claim that may be made by its manufacturer, is not guaranteed or endorsed by the publisher.
