# Supplementary material for: Microbial Dispersal, Including Bison Dung Vectored Dispersal, Increases Soil Microbial Diversity in a Grassland Ecosystem
Source: Front Microbiol. 2022 Mar 31;13:825193. doi: 10.3389/fmicb.2022.825193 (PMC9009311; doi:10.3389/fmicb.2022.825193)
Supplement: Supplementary file 1 [file Table_1.DOCX]

Supplementary Material

# Supplementary Data

There is no supplementary data.

# Supplementary Figures and Tables

There are two supplementary figures.

## Supplementary Figures


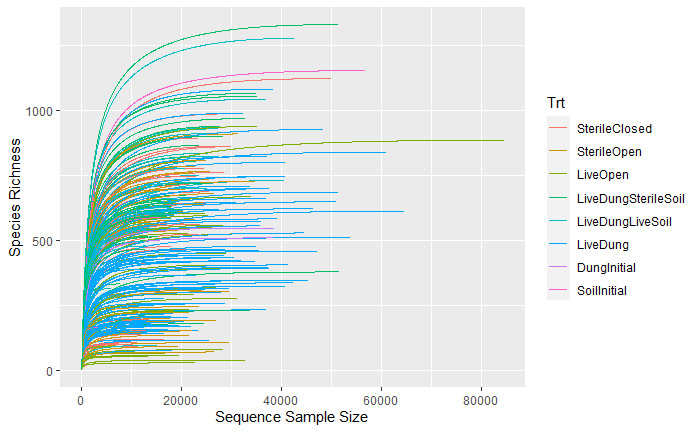

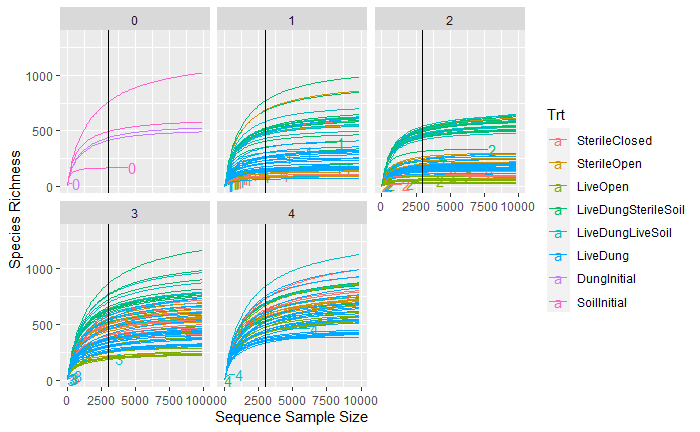


**A**

**B**

**Supplementary Figure 1.** Rarefaction curves for (A) all data and (B) all data faceted by time and only displaying to 10,000 sequences with a vertical line at 3000 sequences.

**A**

**B**


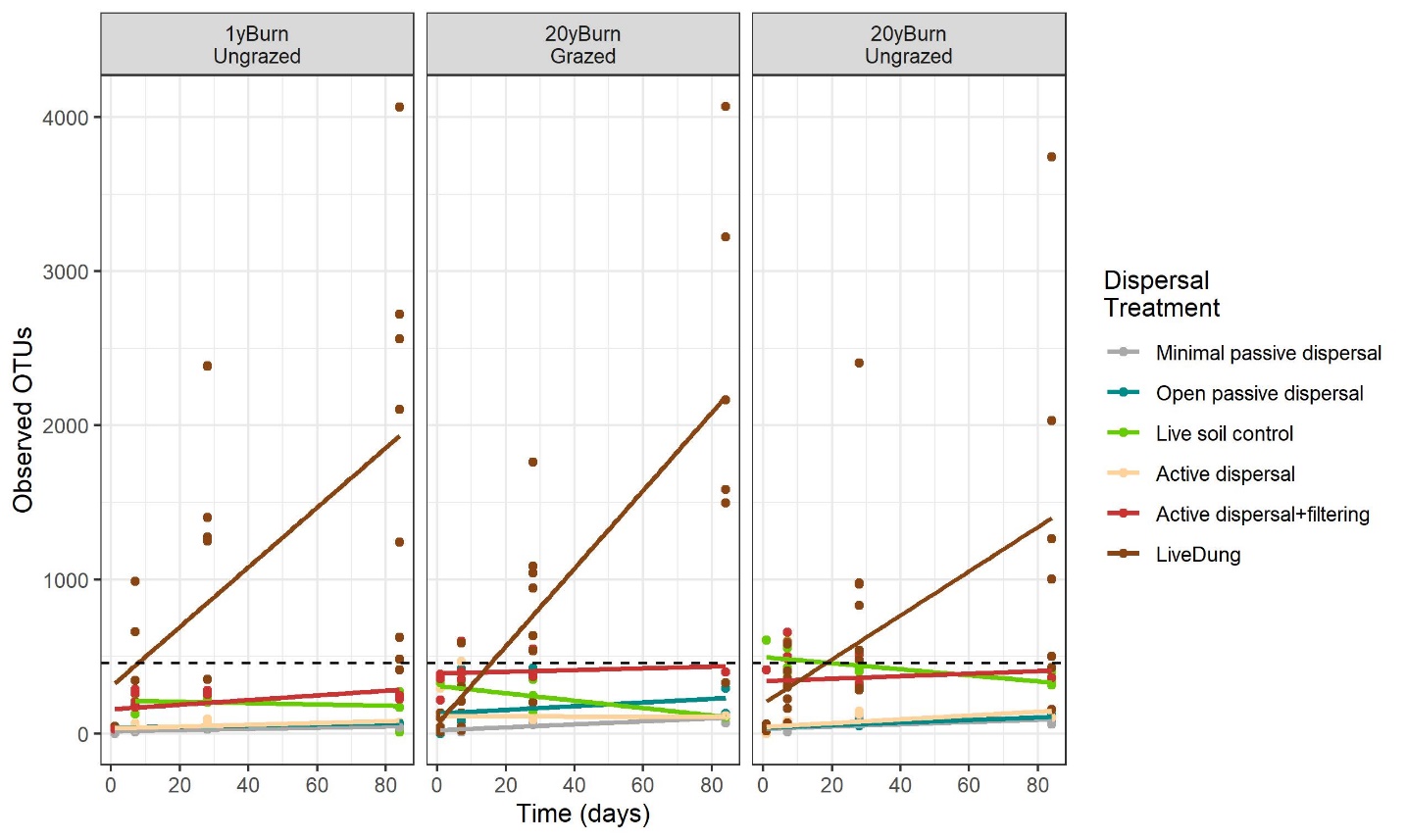

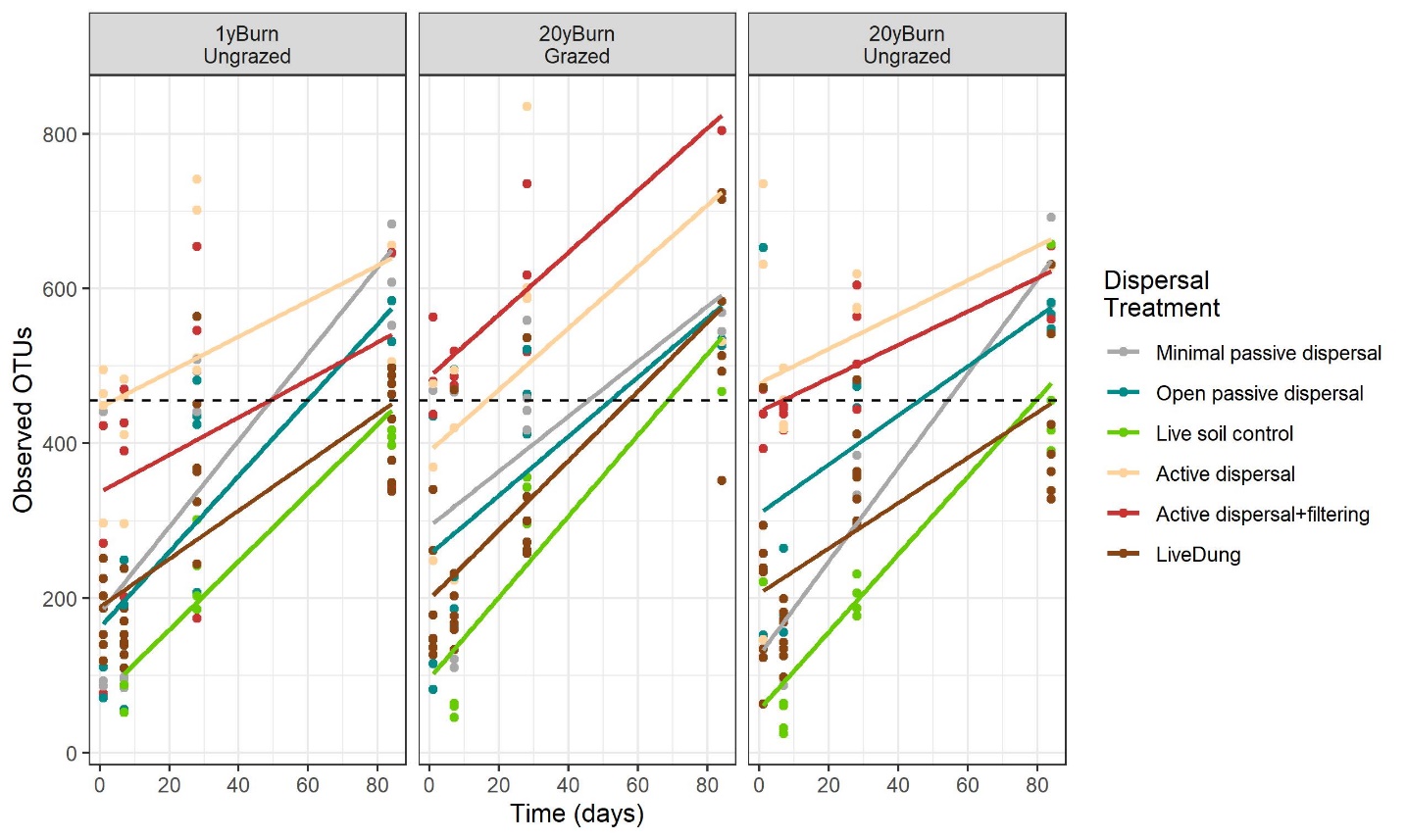


**Supplementary Figure 2.** (A) DNA yield (ng g-1 dry substrate) across time and (B) microbial richness (observed OTUs) across time faceted by land use with reference soil richness indicated by black dashed line.
