# Supplementary material for: Microbial Dispersal, Including Bison Dung Vectored Dispersal, Increases Soil Microbial Diversity in a Grassland Ecosystem
Source: Front Microbiol. 2022 Mar 31;13:825193. doi: 10.3389/fmicb.2022.825193 (PMC9009311; doi:10.3389/fmicb.2022.825193)
Supplement: Supplementary file 3 [file Table_3.DOCX]

---

title: "Frontiers Manuscript Code"

author: "Jaide Hawkins"

date: "10/25/2021"

output: html_document

---

```{r setup, include=FALSE}

knitr::opts_chunk$set(echo = TRUE)

```

## Manuscript Analysis

This document provides the code to perform statistical analysis and create graphs and tables reported in the manuscript. The initial bioinformatics was performed in QIIME2, but the three necessary files have been made freely available. The feature table was filtered to remove positive control features, positive and negative samples, blanks samples, and mitochondria and chloroplast features. The files were formatted and converted to .csv files before proceeding.

Files needed:

- OTU table (i.e feature table, made from BIOM file in QIIME, but will be imported as a .csv); file name: 'OTU_feature_table.csv'

- classified taxonomy; file name: 'OTU_taxonomy.csv'

- sample metadata; file name = SampleData.xlsx. This file contains two tabs so before loading into R, be sure to export the tab named 'Sample Metadata' as a separate Excel file and name it 'Exp_R_master.xlsx'

```{r, libs}

#might need to install packages because many of these are contingent on others

# go here to get installation instructions for phyloseq https://joey711.github.io/phyloseq/install.html

library(phyloseq)

library(ggplot2)

library(ggrepel)

#devtools::install_github("gauravsk/ranacapa")

library(ranacapa) #to visualize rarefaction curves/ rarefying at different depths

library(dplyr)

library(tibble)

library(readxl)

library(vegan)

library(codyn)

library(car)

library(lsmeans)

library(multcomp)

library(multcompView)

library(RColorBrewer)

library(pals)

```

#### Read in data and filter the sample metadata to match the samples in the feature table

The OTU table was made in QIIME and has the positive features removed, blanks and negative/positive samples removed, and mitochondria/chloroplast features removed.

The Tax table is pulled from a zipped folder created in QIIME (a .qza)

The sample data, is the metadata providing treatment information and DNA concentration and yield for each sample.

```{r, data}

#Load data and check head

OTU <- read.csv("OTU_feature_table.csv")

head(OTU, 3)

Tax <- read.csv("OTU_taxonomy.csv")

head(Tax, 3)

Samples_df <-read_excel("Exp_R_master.xlsx")

head(Samples_df, 3)

str(Samples_df)

```

Make the metadata and OTU feature table have the same samples. Since the feature table has less samples than the metadata, filter the metadata based on the samples found in the feature table.

```{r, matching}

#make a vector of samples to keep from feature table

samples_keep <- colnames(OTU[,-1]) # -1 is to remove 'OTUs" from list

head(samples_keep)

Samples_df_filt <- Samples_df %>% filter(`Sample-ID` %in% samples_keep) %>% as.data.frame()

str(Samples_df_filt)#make sure its a data.frame, sample_data() is expecting a df

#changing some variables to factors

factor_cols <- c("watershed", "LandUse", "TimePoint", "Trt", "graze", "burn", "dung")

Samples_df_filt[factor_cols] <- lapply(Samples_df_filt[factor_cols], as.factor)

Samples_df_filt$Trt <- factor(Samples_df_filt$Trt, levels = c("SterileClosed", "SterileOpen", "LiveOpen", "LiveDungSterileSoil", "LiveDungLiveSoil", "LiveDung", "DungInitial", "SoilInitial"))

#changing some varialbles to numeric

numeric_cols <- c("Days", "DNA_conc", "DNAyield")

Samples_df_filt[numeric_cols] <- lapply(Samples_df_filt[numeric_cols], as.numeric)

#check again

str(Samples_df_filt)

```

Will use the 'Samples_df_filt' dataframe from now on.

### Create phyloseq objects

First define row names and then drop old row name

```{r}

#define row names

row.names(OTU) <- OTU$OTUs

OTU <- OTU %>% dplyr::select(-OTUs) #remove old row name

#now for the other data frames

row.names(Tax) <- Tax$Feature.ID

Tax <- Tax %>% dplyr::select(-'Feature.ID')

row.names(Samples_df_filt) <- Samples_df_filt$`Sample-ID`

Samples_df_filt <- Samples_df_filt %>% dplyr::select(-'Sample-ID')

```

OTU table and taxonomy need to be transformed to matrices, sample info left as dataframe

```{r}

OTU <- as.matrix(OTU)

Tax <- as.matrix(Tax)

```

Then transform to phyloseq objects and visualize data

```{r, phylo object}

OTU_phylo <- otu_table(OTU, taxa_are_rows = T)

Tax_phylo <- tax_table(Tax)

samples <- sample_data(Samples_df_filt)

exp0 <- phyloseq(OTU_phylo, Tax_phylo, samples)

exp0

```

### Pre-processing

1. **Prune data to remove taxa that may be sequencing artifacts.**

A. remove samples that do not have a meet minimum reads

B. remove taxa that do not have minimum abundance per samples

Visualize data

```{r, vis sequences}

sample_names(exp0)

rank_names(exp0)

sample_variables(exp0)

sample_sums(exp0)

#checking for empty taxa or samples

any(taxa_sums(exp0)==0)

any(sample_sums(exp0)==0)

#looking at reads per sample

readsumsdf = data.frame(nreads = sort(taxa_sums(exp0), TRUE), sorted = 1:ntaxa(exp0),

type = "OTUs")

readsumsdf = rbind(readsumsdf, data.frame(nreads = sort(sample_sums(exp0),

TRUE), sorted = 1:nsamples(exp0), type = "Samples"))

title = "Total number of reads"

p = ggplot(readsumsdf, aes (x = sorted,y = nreads)) + geom_bar(stat = "identity")

p + ggtitle(title) + facet_wrap(~type, 1, scales = "free")

#looking at reads per sample zoomed in

readsumsdf = data.frame(nreads = sort(taxa_sums(exp0), TRUE), sorted = 1:ntaxa(exp0),

type = "OTUs")

readsumsdf = rbind(readsumsdf, data.frame(nreads = sort(sample_sums(exp0),

TRUE), sorted = 1:nsamples(exp0), type = "Samples"))

title = "Total number of reads"

p = ggplot(readsumsdf, aes (x = sorted,y = nreads)) + geom_bar(stat = "identity")

p + ggtitle(title) + facet_wrap(~type, 1, scales = "free") + ylim(0,10000)

#to view with it colored by trt

#make data frame with total reads per sample and metadata

exp0_data <- data.frame(sample_data(exp0))

exp0_reads <- as.data.frame(sample_sums(exp0))

exp_reads_data <- bind_cols(exp0_data, exp0_reads)

ggplot(exp_reads_data) + geom_point(aes(DNA_conc, exp_reads_data$`sample_sums(exp0)`))

```

Explore rarefaction by using the function ggrare in ranacapa to visual the impact of different sampling depths on richness grouped by Trt. The last line of code is to make Figure S1.

```{r}

exp0

#remove sample 14, 21, 214, 221, 325, and 36 because they only have 1 read, only for this function, will not used this pruned object for anything else

exp0_rare <- prune_samples(sample_sums(exp0)>2, exp0)

exp0_rare #to check that it removed 6 samples

#faceted by time point and x limit of 5000 sequence to zoom in. add vertical line at 3000 which is what I rarefied to

ggrare(exp0_rare, step = 100, color = "Trt", label = "TimePoint", se = FALSE) + facet_wrap(~TimePoint) + xlim(0,10000) + geom_vline(xintercept = 3000)

```

Remove samples that do not meet minimum reads. The last few lines of code are to see which samples were cut.

```{r, minReads}

#look at the library without removing any samples

exp0

## remove the # from the beginning to explore other rarefaction levels

#exp_2000 <- prune_samples(sample_sums(exp0)>2000, exp0)

#exp_2000 #removed 39 samples - 12.5%

exp_3000 <- prune_samples(sample_sums(exp0)>3000, exp0)

exp_3000 #removed 41 samples - 13.1%

#exp_5000 <- prune_samples(sample_sums(exp0)>5000, exp0)

#exp_5000 #removed 49 samples - 15.7%

#exp_10000 <- prune_samples(sample_sums(exp0)>10000, exp0)

#exp_10000 #removed 58 samples - 18.5%

#rename to exp 1

exp1 <- exp_3000

#to see which samples did not make the cut

OrgSamples <- data.frame(samples=sample_names(exp0))

MinReadsSamples <- data.frame(samples=sample_names(exp1))

droppedSamples <- anti_join(OrgSamples, MinReadsSamples, by = "samples") #anti-join shows which samples in OrgSamples do not have a match in the new cut data set

droppedSamples

```

Remove OTUs that do not have at least 5 reads/sample.

```{r, prune OTUs}

#using exp1 (samples with a at least 3000 reads)

exp1

wh0 = genefilter_sample(exp1, filterfun_sample(function(x) x > 5))

exp_prune <- prune_taxa(wh0, exp1)

exp_prune# removed 9049 OTUs - does this seem high?

sum(taxa_sums(exp_prune))

```

2. **create rarefy and normalize objects**

using exp_prune for this (sample size of at least 3000)

normalize # of reads in each sample using Total Sum Scaling (TSS) normalization: this method removes technical bias related to different sequencing depth in different libraries via simply dividing each feature count with the total library size to yield relative proportion of counts for that feature. For easier interpretation, we can multiply it by 1,000,000 to get the number of reads corresponding to that feature per million reads (definition from [this site](https://www.microbiomeanalyst.ca/MicrobiomeAnalyst/docs/FaqView.xhtml#norm2). Provides other normalization methods)

Setting a seed before rarifying and normalizing ensures reproducible data.

The end of this code chunk is to find the most abundant phyla and top 10 taxa within the normalized dataset and RA of other important phyla

```{r, rare and norm}

set.seed(400)

expr <- rarefy_even_depth(exp_prune, sample.size = 3000) #63 OTUs removed

#normalize

set.seed(300)

expn <- transform_sample_counts(exp_prune, function(x) 10000 * x/sum(x))

#look at each library and find total number of sequences for each

exp_prune

sum(taxa_sums(exp_prune))

expr

sum(taxa_sums(expr))

expn

sum(taxa_sums(expn))

par(mfrow = c(1, 2))

title = "Sum of reads for each sample, expr"

plot(sort(sample_sums(expr), TRUE), type = "h", main = title, ylab = "reads",

ylim = c(0, 10000))

title = "Sum of reads for each sample, expn"

plot(sort(sample_sums(expn), TRUE), type = "h", main = title, ylab = "reads",

ylim = c(0, 10000))

```

### Alpha Diversity

using the rarified dataset: expr

This section to addresses the first sub hypotheses related to changes in richness over time within the watersheds to examine dispersal differences among land use treatment.

Plot overall richness for all samples then make a dataframe with the richness values for each sample matched up with the metadata. This new dataframe will be used to make some of the graphs in the manuscript. Called 'exp_richness_df'

```{r, alpha DF}

###overall alpha diversity plots

##this are not used in the manuscript, but good exploratory plots

##remove # to view

#plot_richness(expr, measures = c("Observed", "Shannon"), color = "Trt") + theme(axis.text.x = element_text(angle = 90)) + ggtitle("by Treatment")

#(expr, measures = c("Observed", "Shannon"), color = "LandUse") + theme(axis.text.x = element_text(angle = 90)) + ggtitle("by LandUse")

#plot_richness(expr, measures = c("Observed", "Shannon"), color = "TimePoint") + theme(axis.text.x = element_text(angle = 90)) + ggtitle("by TimePoint")

#values for each sample

exp_richness <- estimate_richness(expr, split = TRUE, c("Observed", "Chao1", "Shannon"))

row.names <- row.names(sample_data(expr))

row.names <- as.data.frame(row.names)

names(row.names)[1] <- "Sample-ID"

#now add the sample ID column, can bind cause I know its in the same order as the dataframe it was derived from

exp_richness <- bind_cols(exp_richness, row.names)

head(exp_richness, 3)

#adding sample code, site and graze columns to export file

sample_info <- data.frame(sample_data(expr)) %>% tibble::rownames_to_column(var = "Sample-ID")

head(sample_info, 3)

#now join the sample info with the newly calculated richness stats

exp_richness_df <- full_join(sample_info, exp_richness, by = "Sample-ID")

#to add a new variable

#exp_richness_df <- exp_richness_df %>% transform(NewVar = paste(Var1, Var2))

#exp_richness_df$NewVar <- as.factor(exp_richness_df$NewVar)

head(exp_richness_df)

str(exp_richness_df)

```

Need to find the mean number of observed OTUs for Soil initials and live dung to add to richness plots -- will use these when graphing dispersal rate.

```{r, initial mean OTUs}

#Soil

InitialSoilRich <- exp_richness_df %>% filter(Trt == "SoilInitial") %>% summarise(avg.observed = mean(Observed))

InitialSoilRich

InitialSterileDung <- exp_richness_df %>% filter(Trt == "DungInitial") %>% summarise(avg.observed = mean(Observed))

InitialSterileDung

```

Average initial soil = 455

Average initial dung = 414

The next code chunk to to make Manuscript Figures 3 and Supplemental 2. They are DNA yield and richness plotted over time and grouped by treatment.

```{r, richness}

#by treatment

#########all data graphed together and not faceted by land use -- for figure 3 A and B

#first filter out dung and soil initials

exp_richness_trts <- exp_richness_df %>% dplyr::filter(Trt %in% c("SterileClosed", "SterileOpen", "LiveOpen", "LiveDungSterileSoil", "LiveDungLiveSoil", "LiveDung"))

#create color palette

trtColors_noInitials = c("darkgray", "darkcyan", "chartreuse3", "burlywood1", "brown3", "chocolate4")

####OTUs richness

jpeg("dispersal_fullScatter.jpeg", width = 8.5, height = 5, units = 'in', res = 350)

ggplot(exp_richness_trts, aes(Days, Observed, color = Trt)) + geom_point() +

geom_smooth(aes(group = Trt),method = "lm", se = FALSE) +

theme_bw() + xlab("Time (days)") + ylab("Observed OTUs") +

geom_hline(yintercept = 455, linetype = "dashed", color = "black", show.legend = TRUE) +

scale_color_manual(values = trtColors_noInitials, name = "Dispersal\nTreatment",

labels = c("Minimal passive dispersal", "Open passive dispersal", "Live soil control", "Active dispersal", "Active dispersal+filtering", "LiveDung"))

####DNA yield

jpeg("DNAyield_fullScatter.jpeg", width = 8.5, height = 5, units = 'in', res = 350)

ggplot(exp_richness_trts, aes(Days, DNAyield, color = Trt)) + geom_point() +

geom_smooth(aes(group = Trt),method = "lm", se = FALSE) +

theme_bw() + xlab("Time (days)") + ylab("log(DNA yield (ng DNA/g dry substrate))") +

scale_y_log10() +

scale_color_manual(values = trtColors_noInitials, name = "Dispersal\nTreatment",

labels = c("Minimal passive dispersal", "Open passive dispersal", "Live soil control", "Active dispersal", "Active dispersal+filtering", "LiveDung"))

####################################### scatterplots of richness by time grouped by Trt and faceted by land use separate for each land use, for Figure S2

## I will use the already subsetted df 'exp_richness_trts' which does not contain the initial samples

#here are the treatment colors again

trtColors = c("darkgray", "darkcyan", "chartreuse3", "burlywood1", "brown3", "chocolate4", "black", "deeppink1")

#create character vector for renaming facet labels

land.labels <- c("1yBurn\nUngrazed", "20yBurn\nGrazed", "20yBurn\nUngrazed")

names(land.labels) <- c("B_UG", "UB_G", "UB_UG")

############richness/ dispersal rate

jpeg("linegraphs_dispersal_allTrts_supp.jpeg", width = 8.5, height = 5, units = 'in', res = 300)

ggplot(exp_richness_trts, aes(Days, Observed, color = Trt)) + geom_point() +

geom_smooth(aes(group = Trt),method = "lm", se = FALSE) + facet_wrap(~LandUse, labeller = labeller(LandUse = land.labels)) +

theme_bw() + xlab("Time (days)") + ylab("Observed OTUs") +

geom_hline(yintercept = 455, linetype = "dashed", color = "black", show.legend = TRUE) +

scale_color_manual(values = trtColors, name = "Dispersal\nTreatment", labels = c("Minimal passive dispersal", "Open passive dispersal", "Live soil control", "Active dispersal", "Active dispersal+filtering", "LiveDung"))

###########DNA concentration

jpeg("linegraphs_DNA_allTrts_supp.jpeg", width = 8.5, height = 5, units = 'in', res = 300)

ggplot(exp_richness_trts, aes(Days, DNAyield, color = Trt)) + geom_point() +

geom_smooth(aes(group = Trt),method = "lm", se = FALSE) + facet_wrap(~LandUse, labeller = labeller(LandUse = land.labels)) +

theme_bw() + xlab("Time (days)") + ylab("Observed OTUs") +

geom_hline(yintercept = 455, linetype = "dashed", color = "black", show.legend = TRUE) +

scale_color_manual(values = trtColors, name = "Dispersal\nTreatment", labels = c("Minimal passive dispersal", "Open passive dispersal", "Live soil control", "Active dispersal", "Active dispersal+filtering", "LiveDung"))

```

#### ANCOVAS

Need to compare the rate of dispersal, i.e the number of new OTUs added over time among the three treatments within land uses and between land uses. I will be using the lm function to create model with interaction, then using the Anova function from the car package to run the ANCOVA. If the interaction is significant then the slopes are statistically different. If only the main effects are significant then observed OTUs changes across time and is different among trts but that rate (accumulation of OTUs) does not depend on trt.

**Full ANOVAs**

Not split up by land use

```{r}

#ANOVA for richness all trts

rich.full.lm <- lm(Observed ~ Trt*LandUse, data= rich.noInitials)

anova(rich.full.lm)

summary.lm(rich.full.lm)

lsmeans(rich.full.lm, pairwise~Trt, adjust="tukey")

#to get letters for post hoc groups

Pairs_rich <- glht(rich.full.lm, linfct = mcp(Trt = "Tukey"))

print(summary(Pairs_rich)) # pairwise tests

cld(Pairs_rich) # compact letter display

#ANOVA for DNA all trts

DNA.full.lm <- lm(DNAyield ~ Trt*LandUse, data= rich.noInitials)

anova(DNA.full.lm)

summary.lm(DNA.full.lm)

lsmeans(DNA.full.lm, pairwise~Trt, adjust="tukey")

#to get letters for post hoc groups

Pairs_DNA <- glht(DNA.full.lm, linfct = mcp(Trt = "Tukey"))

print(summary(Pairs_DNA)) # pairwise tests

cld(Pairs_DNA) # compact letter display

```

**FULL ANCOVAS**

From the first full model ancova ran, land use is not significant, and therefore not used as factor in reduced models. These are the stats are used in table 1 and 2.

```{r, full ANCOVAS}

####################################################################### Richenss ANCOVAs

##########full model

rich.full.ancova <- lm(Observed~Days*Trt*LandUse, data = rich.noInitials)

car::Anova(rich.full.ancova, type = "III")

summary(rich.full.ancova)

rich.full.ancova.lsmeans <- lsmeans(rich.full.ancova, ~Trt*LandUse)

print(cld(rich.full.ancova.lsmeans,

alpha=0.05,

Letters=letters,

adjust = "tukey"))

#obtain slopes

rich.full.ancova$coefficients

full.lst.slopes <- lstrends(rich.full.ancova, "Trt", var="Days")

full.lst.slopes

#compare slopes

pairs(full.lst.slopes)

######taking out land use because it was not significant alone nor sig interaction with time

rich.full.ancova2 <- lm(Observed~Days*Trt, data = rich.noInitials)

car::Anova(rich.full.ancova2, type = "III")

summary(rich.full.ancova2) #sterile closed is the reference here, for main interactions the y -intercept is found by adding to the main intercept and slope is calculated by adding values after interaction terms to days

rich.full.ancova2.lsmeans <- lsmeans(rich.full.ancova2, ~Trt)

print(cld(rich.full.ancova2.lsmeans,

alpha=0.05,

Letters=letters,

adjust = "tukey"))

#obtain slopes

rich.full.ancova2$coefficients

#create table to more easily obtain slopes

full.lst.slopes2 <- lstrends(rich.full.ancova2, "Trt", var="Days")

full.lst.slopes2

#compare slopes

pairs(full.lst.slopes2)

##############################################################################Group comparison ANCOVAS (Table 2)

#####Table 2 first subset "richness_SterileSoil" : "SterileClosed", "SterileOpen"

richness_SterileSoil <- exp_richness_df %>% filter(Trt %in% c("SterileClosed", "SterileOpen"))

rich.SterileSoil.ancova <- lm(Observed~Days*Trt*LandUse, data= richness_SterileSoil)

car::Anova(rich.SterileSoil.ancova, type = "III") #these are the values in Table 1

rich.SterileSoil.ancova.lsmeans <- lsmeans(rich.SterileSoil.ancova, ~Trt)

print(cld(rich.SterileSoil.ancova.lsmeans,

alpha=0.05,

Letters=letters,

adjust = "tukey"))

#to get coefficients, take out interaction term

rich.SterileSoil.ancova2 <- lm(Observed~Days*Trt, data= richness_SterileSoil)

car::Anova(rich.SterileSoil.ancova2, type = "III")

summary(rich.SterileSoil.ancova2)

lsmeans(rich.SterileSoil.ancova2, pairwise~Trt, adjust="tukey")

#obtain slopes

rich.SterileSoil.ancova2$coefficients

rich.SterileSoil.lst.slopes2 <- lstrends(rich.SterileSoil.ancova2, "Trt", var="Days")

rich.SterileSoil.lst.slopes2

#compare slopes

pairs(rich.SterileSoil.lst.slopes2)

####table 2 second subset, Open Passive vs. Active Dispersal

richness_SoilandDung_4levels <- exp_richness_df %>% filter(Trt %in% c("SterileOpen", "LiveOpen", "LiveDungSterileSoil", "LiveDungLiveSoil"))

rich.SoilandDung.ancova <- lm(Observed~Days*Trt*LandUse, data= richness_SoilandDung_4levels)

car::Anova(rich.SoilandDung.ancova, type = "III")#these are the values in Table 1

rich.SoilandDung.ancova.lsmeans <- lsmeans(rich.SoilandDung.ancova, ~Trt) #not including land use because it was not signficant

print(cld(rich.SoilandDung.ancova.lsmeans,

alpha=0.05,

Letters=letters,

adjust = "tukey"))

summary.lm(rich.SoilandDung.ancova)

lsmeans(rich.SoilandDung.ancova, pairwise~Trt, adjust="tukey")

####table 2 third subset, Live Dung vs. Active Dispersal. Using richness_onlyDungTrts df ("LiveDungSterileSoil", "LiveDungLiveSoil", and "LiveDung)

rich.Dung.ancova <- lm(Observed~Days*Trt*LandUse, data= richness_onlyDungTrts)

car::Anova(rich.Dung.ancova, type = "III")#these are the values in Table 1

rich.Dung.ancova.lsmeans <- lsmeans(rich.Dung.ancova, ~Trt) #not including land use because it was not signficant

print(cld(rich.Dung.ancova.lsmeans,

alpha=0.05,

Letters=letters,

adjust = "tukey"))

summary(rich.Dung.ancova)

############################################################################### DNA ANCOVAs #########################################################

#FULL model

DNA.full.ancova <- lm(DNAyield~Days*Trt*LandUse, data = rich.noInitials)

car::Anova(DNA.full.ancova, type = "III")

summary(DNA.full.ancova)

DNA.full.ancova.lsmeans <- lsmeans(DNA.full.ancova, ~Trt*LandUse)

print(cld(DNA.full.ancova.lsmeans,

alpha=0.05,

Letters=letters,

adjust = "tukey"))

#obtain slopes

DNA.full.ancova$coefficients

full.DNA.lst.slopes <- lstrends(DNA.full.ancova, "Trt", var="Days")

full.DNA.lst.slopes

#compare slopes

pairs(full.DNA.lst.slopes)

###taking out land use full model (What was used for Table 2)

DNA.full.ancova2 <- lm(DNAyield~Days*Trt, data = rich.noInitials)

#check assumptions

qqnorm(DNA.full.ancova2$residuals)

plot(DNA.full.ancova2$fitted.values, DNA.full.ancova2$residuals, col = "blue")

car::Anova(DNA.full.ancova2, type = "III")

summary(DNA.full.ancova2)

DNA.full.ancova2.lsmeans <- lsmeans(DNA.full.ancova2, ~Trt, adjust = "Tukey")

print(cld(DNA.full.ancova2.lsmeans,

alpha=0.05,

Letters=letters,

adjust = "tukey"))

#obtain slopes

DNA.full.ancova2$coefficients

full.DNA.lst.slopes2 <- lstrends(DNA.full.ancova2, "Trt", var="Days")

full.DNA.lst.slopes2

#compare slopes

pairs(full.DNA.lst.slopes2)

####see next code chunk for ind. regression lines

####################Treatment Subset comparisons

###Table 2 first subset "richness_SterileSoil" : "SterileClosed", "SterileOpen"

DNA.noDungTrts.ancova <- lm(DNAyield~Days*Trt*LandUse, data= richness_SterileSoil)

car::Anova(DNA.noDungTrts.ancova, type = "III")

#post hoc comparisons

DNA.noDungTrts.ancova.lsmeans <- lsmeans(DNA.noDungTrts.ancova, ~Trt*LandUse)

print(cld(DNA.noDungTrts.ancova.lsmeans,

alpha=0.05,

Letters=letters,

adjust = "tukey"))

DNA.noDungTrts.ancova2 <- lm(DNAyield~Days*Trt, data= richness_SterileSoil)

summary(DNA.noDungTrts.ancova2)

####table 2 second subset, Open Passive vs. Active Dispersal

DNA.SoilandDung.ancova <- lm(DNAyield~Days*Trt*LandUse, data= richness_SoilandDung_4levels)

car::Anova(DNA.SoilandDung.ancova, type = "III")

DNA.SoilandDung.ancova.lsmeans <- lsmeans(DNA.SoilandDung.ancova, ~Trt*LandUse)

print(cld(DNA.SoilandDung.ancova.lsmeans,

alpha=0.05,

Letters=letters,

adjust = "tukey"))

DNA.SoilandDung.ancova.lsmeans2 <- lsmeans(DNA.SoilandDung.ancova, ~Trt)

print(cld(DNA.SoilandDung.ancova.lsmeans2,

alpha=0.05,

Letters=letters,

adjust = "tukey"))

summary(DNA.SoilandDung.ancova)

####table 2 third subset, Live Dung vs. Active Dispersal. Using richness_onlyDungTrts df ("LiveDungSterileSoil", "LiveDungLiveSoil", and "LiveDung)

DNA.Dung.ancova <- lm(DNAyield~Days*Trt*LandUse, data= richness_onlyDungTrts)

car::Anova(DNA.Dung.ancova, type = "III")

DNA.Dung.ancova.lsmeans <- lsmeans(DNA.Dung.ancova, ~Trt) #not including land use because it was not signficant

print(cld(DNA.Dung.ancova.lsmeans,

alpha=0.05,

Letters=letters,

adjust = "tukey"))

summary(DNA.Dung.ancova)

```

**Individual Regression Models for each Treatment (Table 1)**

Regression lines for each dispersal treatment pooled across land use. This is the code chunk used to create Table 1.

```{r, dispersal rate regressions}

#first do across watersheds since land use was not significant without initials

#Richness

dispersalModels <- plyr::dlply(rich.noInitials, "Trt", function(df)

lm(Observed ~ Days, data = df))

# Apply coef to each model and return a data frame

plyr::ldply(dispersalModels, coef)

# Print the summary of each model

plyr::l_ply(dispersalModels, summary, .print = TRUE) #I know the order from the full summary ANCOVA and checking slopes: SterileClosd, SterileOpen, LiveOpen, LiveDungSterileSoil, LiveDungLiveSoil,LiveDung)

#DNA yield

DNAyieldModels <- plyr::dlply(rich.noInitials, "Trt", function(df)

lm(DNAyield ~ Days, data = df))

# Apply coef to each model and return a data frame

plyr::ldply(DNAyieldModels, coef)

# Print the summary of each model

plyr::l_ply(DNAyieldModels, summary, .print = TRUE) #I know the order from the full summary ANCOVA and checking slopes

```

### NMDS and Beta diversity

First do multivariate stats using Bray-Curtis Distance and NMDS:

```{r}

#perform ordination using phyloseq function

exp.ord <- ordinate(expn, "NMDS", "bray")

exp.ord

```

Then plot ordination

```{r, NMDS}

#create color scheme of 9 colors

trtColors = c("darkgray", "darkcyan", "chartreuse3", "burlywood1", "brown3", "chocolate4", "black", "deeppink1")

jpeg("samplesNMDS_Trt_Time.jpeg", width = 8.5, height = 5, units = 'in', res = 300)

plot_ordination(expn, exp.ord, type = "samples", color = "Trt", shape = "TimePoint") +

scale_color_manual(values=trtColors, na.translate = FALSE, name = "Dispersal\nTreatment", labels = c("Minimal passive dispersal", "Open passive dispersal", "Live soil control", "Active dispersal", "Active dispersal+filtering", "LiveDung", "Dung Reference", "Soil Reference")) +

scale_shape_manual(values = c(8, 16, 17, 15, 5), na.translate = FALSE, name = "Time Point", label = c("0 = Reference", "1 = 1 day", "2 = 7 days", "3 = 28 days", "4 = 84 days")) + geom_point(size=2) +

theme_bw()

```

### PERMANOVA and dispersion in Vegan

First make a distance object using Bray Curtis dissimilarity. Using phyloseq, but it is actually calling to the Vegan package vegdist function.

```{r, bray}

reg_bray <- phyloseq::distance(expn, "bray")

```

Then perform the PERMANOVA by specifying formula and data frame the predictors are in. Used the exp.env df from above and performed 3-way PERMONANOVA with Trt, TimePoint, and LandUse as my factors. Use an * in the formula to test interaction of the three factors.

This chunk also runs a dispersion analysis using betadisper() in vegan and makes Figure 5. A new variable that is time and treatment combined was created since betadisper() cannot specify two different factors.

```{r, perm}

####################################################################################### PERMANOVA

set.seed(58)

adonis(reg_bray ~ Trt*TimePoint*LandUse, data = exp.env, permutations = 999)

####################################################################################### DISPERSION

#create new time_Trt variable.

exp.env$Time_Trt <- as.factor(paste(exp.env$TimePoint, exp.env$Trt))

str(exp.env)

#and now run function to find dispersion within treatments

Trt.dispersion <- betadisper(reg_bray, group = exp.env$Time_Trt)

Trt.dispersion

anova(Trt.dispersion)

permutest(Trt.dispersion, pairwise = TRUE, permutations = 999)

#join the distances with the environmental data so I can graph

exp.env.2 <- exp.env %>% rownames_to_column()

exp.dispersion.values <- as.data.frame(Trt.dispersion$distances) %>% rownames_to_column()

exp.env.distances <- full_join(exp.env.2, exp.dispersion.values, by = "rowname")

#change column name

exp.env.distances$distanceTOcent <- exp.env.distances$`Trt.dispersion$distances`

#filter out uwanted initial trts

exp.env.distances <- exp.env.distances %>% filter(TimePoint %in% c("1", "2", "3", "4"))

#make line graphs using averages for each treatment across time

jpeg("exp.trt.dispersion.jpeg", width = 8.5, height = 5, units = 'in', res = 350)

exp.env.distances %>% group_by_at(vars(TimePoint, Trt)) %>% summarize(mean.distance = mean(distanceTOcent), sd = sd(distanceTOcent)) %>%

ggplot(aes(TimePoint, mean.distance, color = Trt, group = Trt)) + geom_point() + geom_line(size = 1) +

geom_errorbar(aes(ymin = mean.distance - sd, ymax = mean.distance + sd), position=position_dodge(0.1)) +

theme_bw() +

xlab("Time (days)") + ylab("Average Distance to Centroid") +

scale_color_manual(values = trtColors, name = "Dispersal\nTreatment", labels = c("Minimal passive dispersal", "Open passive dispersal", "Live soil control", "Active dispersal", "Active dispersal+filtering", "LiveDung"))

```
